# Demonstrative Midwifery and C. M.

**Published:** 1850-12

**Authors:** 


*Demonstrative Midwifery and C. M.*—In the last number of this Journal, we alluded to the publication of a notice of the trial of Loomis for libel, by a writer in the American Journal of Medical Sciences over the signature of C. M., and we offered to reproduce the article in our columns if desired. We had then given the notice but a casual examination. We have since read it with more attention, and we recur to the subject now, to express astonishment, in the first place, that any person, after reading the report of the trial just mentioned, whatever might be his predilections as regards either the subject or individuals concerned, could give as a fair statement of the facts developed therein, the distorted account contained in the article signed C. M. That account, we aver, contains gross misstatements relative to the demonstration by the Prof. of Obstetrics in the University of Buffalo.

Our astonishment, in the second place, is not less in finding, in the article by C. M., the professional conduct of the Prof. of Obstetrics assailed on the ground of unskilfullness in not rectifying the position of the fœtus in the early stage of labor. We have no fear of standing alone when we avow the opinion, that a writer who ventures deliberately to charge a fellow member of the profession with mal-practice, should be prepared with proof, clear and conclusive, to sustain the accusation. We deny that any such proof is contained in the notice by C. M., or that it is in any measure available in the present instance.

For the present we rest with this affirmation and denial, pledging ourselves, in a future No., if occasion seems to call for it, to make good our assertions, and, at the same time, to consider the validity of the arguments offered by C. M. in opposition to demonstrative instruction in midwifery.

Having offered to copy the article from the Am. Jour. of Med. Sciences, if requested, we should state that no intimation of a wish by any one to that effect has been received.

The Report of the trial has been reviewed by the New Orleans Med. Journal, the Charleston, S. C., Med. Jour., the Northern Lancet, the Louisville Journal, and the New Hampshire Med. Jour., each of which takes a decided stand in support of the course pursued by the Faculty of the Med. College of Buffalo.

